# Optimization design of battery bracket for new energy vehicles based on 3D printing technology

**DOI:** 10.1038/s41598-024-64393-x

**Published:** 2024-06-12

**Authors:** Guoqing Zhang, Xueyan Li, Junxin Li, Xiaoyu Zhou, Yongsheng Zhou

**Affiliations:** School of Mechanical and Electrical Engineering, Zhoukou Normal University, Zhoukou, 466000 Henan People’s Republic of China

**Keywords:** 3D printing, Topology optimization design, Battery pack, Bracket, Geometric reconstruction, Electrical and electronic engineering, Mechanical engineering

## Abstract

Nowadays, what captures consumers' primary attention is how to purchase electric vehicles with long range and desirable price. Lightweight construction stands as one of the most effective approaches for prolonging range and lowering costs. As a consequence, it is particularly imperative to undertake lightweight design optimization for the battery bracket of new energy vehicles by applying 3D printing technology. To actualize this goal, Rhino software was initially employed for 3D modeling to design the battery bracket system for a pure electric vehicle in China. Subsequently, topology optimization design of the battery bracket was carried out by adopting Altair Inspire software. Last but not least, manufacturing and assembly inspection were completed using a 3D printer. The results show that the maximum displacement of the battery lower tray bracket after topology optimization is 3.20 mm, which is slightly higher than before, but still relatively small. The maximum Mises equivalent stress rose to 240.7 MPa post-optimization, but brought about a uniform stress distribution at the bottom of the bracket. In comparison, the minimum factor of safety met design requirements at 1. The mass was lessened to 0.348 kg, representing a 49.2% decrease in comparison with pre-optimization levels. The 3D-printed bracket was fabricated by employing a 3D printer, thereby achieving the aforementioned mass abatement. The battery pack parts exhibited a bright surface with low roughness and no discernible warping or deformation defects. As revealed by the assembly results, the components of the battery pack bracket are tightly coordinated with each other, with no evident assembly conflicts, revealing that the dimensional accuracy and fit of the completed parts meet production requirements. These findings lay solid groundwork for the mass production of high-performance battery pack brackets.

## Introduction

In accordance with statistics from the Traffic Administration of the Ministry of Public Security, up to the end of 2023, the national ownership of new energy vehicles reached 20.41 million, accounting for 6.07% of the automobiles in total. In 2023 alone, 7.43 million new energy vehicles were registered, mirroring a notable year-on-year increase of 38.76%. Serving as the primary component responsible for carrying and protecting the power battery, the battery bracket fulfills paramount roles including battery system support, heat dissipation, collision prevention, and bottom contact prevention. It stands as the most significant large component of new energy vehicles, occupying a pivotal position within the battery pack system^1^. Currently, enterprises utilize aluminum alloy battery brackets, which are severely limited by their heavy weight and high cost. Furthermore, these battery brackets endure heavy loads. Nonetheless, the fatigue performance of aluminum alloy is merely half that of steel, and its modulus of elasticity is only one-third of steel, thereby offering substantial room for optimization in design. As the market demand for battery pack energy density multiplies progressively, particularly in the context of new energy pure electric vehicles, where a 10% diminution in vehicle overall mass brings about a 5.5% decrease in electric power consumption and a 5.5% increase in range, it become increasingly imperative for us to invest more on lightweight design optimization for the battery pack bracket^2^.

3D printing technology involves the utilization of specialized software to slice and layer three-dimensional models, generating cross-sectional data which is subsequently imported into rapid prototyping equipment. This technology employs a layer-by-layer approach to fabricate solid parts. Thanks to this additive manufacturing method, 3D technology can effectively produce components of virtually any geometric shape. Its advantages include the ability to process single pieces or small batches, accommodate complex geometric structures, and achieve dense parts organization^3–5^. Leveraging the aforementioned advantages of 3D printing technology, its application in the development of new energy electric vehicle battery pack brackets holds significant promise for expediting the development cycle and reducing associated costs.

Tang et al.^6^ validated the precision of the side column collision finite element model of the power battery system through vehicle side column collision testing. They probed deep into the deformation state and acceleration response of the battery pack under the collision scenario involving rigid side columns. As displayed by their research findings, during side column collisions, both the outer frame and internal support structure of the power battery pack experienced striking deformation, while the battery system encountered substantial acceleration impacts across all positions. Zheng^7^ adopted finite element analysis software to conduct lightweight design optimization of a specific brand's new energy vehicle battery pack enclosure. It’s noteworthy that their optimized case's weight decreased from 110.56 kg to 62.74 kg, which materialized a light-weighting rate of 43.25%. Furthermore, the displacement, stress, and strain of the optimized case fell within the prescribed design parameters, which highly aligns with the design requirements. In the studies carried out by Zhang et al.^8^, the structure of the battery pack has been optimized to mitigate the stress and deformation arisen from external forces on the high-voltage battery pack. They validated the optimization results through simulation analysis. Following optimization, the maximum stress and deformation experienced by the battery pack were strikingly lowered, giving rise to an augment in the crash safety of the battery pack. Gao et al.^9^ first took the power pack of a certain type of pure electric vehicle as their research object. Based upon the Optistruct software, the topology optimization design of the module end plates was carried out correspondingly, which triggered a weight reduction of 11.56 kg with a weight abatement ratio of 31.88%. Then the third-order response surface approximation model was solved by multi-objective optimization by referring to the non-dominated sorting genetic algorithm with elite strategy (NSGA-II), which gave rise to a weight reduction of 3.66 kg, with a weight abatement ratio of 9.03%, and the total weight reduction of the power pack was 15.22 kg, with a total weight abatement ratio of 19.82%, which materializes the anticipated purpose of light-weighting. Jin et al.^10^ employed 6063-T6 aluminum alloy extruded profiles as the primary material for designing the lower housing of the battery pack. They not only completed the structural design of the aluminum alloy battery pack lower shell, but also conducted simulation analysis of the lower shell under load—bearing and extrusion conditions by adopting three-dimensional software modeling. Subsequently, the designed aluminum alloy battery pack lower shell was optimized accordingly in accordance with the results of the simulation analysis. With the purpose of safeguarding the battery pack against bottom-scraping conditions, Wang et al.^11^ put forth a cross-section-arrangement design approach for aluminum alloy protective structures. They developed a simulation model for the entire vehicle's bottom-scraping scenario. As illustrated by their research findings, the ameliorated aluminum alloy protective structure schemes achieved weight abatements of 59.6% and 46.8%, respectively, in comparison with the original steel structure scheme, while ensuring that the intrusion level of the battery module met the specified requirements. In an effort to maximize structural performance while minimizing costs, Amrit et al.^12^ employed data-driven agents and a Multi-Objective Genetic Algorithm (MOGA) to explore battery bracket design. Majid et al.^13^ utilized a genetic algorithm to reinforce the thermal performance of an electric vehicle battery system, bringing about an 8% decrease in the maximum packing temperature and a 16.1% reduction in inter-module temperature variation. With the aim of lessening weight and material costs, Bala et al.^14^ employed topology majorization to redesign the existing battery case of a Volvo vehicle. As a result, the optimized battery pack weight was decreased by approximately 6.3%.

In summary, current scholars have made notable advancements in the design research of new energy electric vehicle battery pack systems, ranging from reinforcing collision safety to reducing overall weight. Nonetheless, there remains vast room for further amelioration, particularly in areas such as achieving additional weight reduction and augmenting the feasibility of batch manufacturing. Consequently, this paper is intended to delve deeper into the potential for performance reinforcement within the battery pack system for new energy electric vehicles.

## Materials and methods

### Design methods

**Figure 1:**
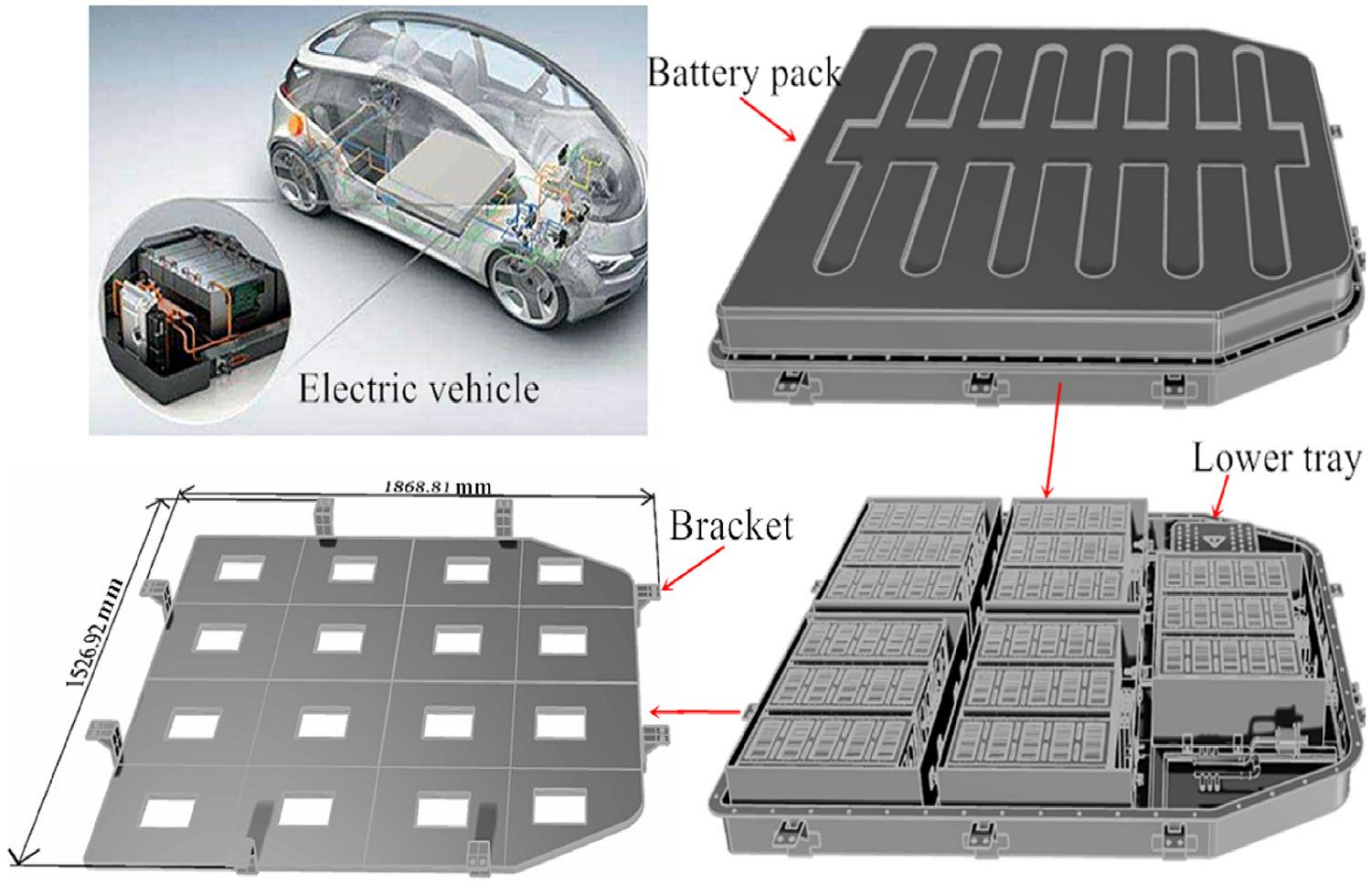
Battery pack system for a certain electric vehicle.

For the time being, light-weighting strategies for battery pack brackets predominantly involve the application of lightweight materials and the implementation of lightweight structural designs. Lightweight material applications for battery pack brackets include the utilization of aluminum alloy, high-strength steel, and composite materials. Among these options, aluminum alloy materials are the mainstream choice as a result of their lightweight properties. Regarding lightweight structural design, considerations such as collision damping, heat dissipation, waterproofing, dust—proofing, and insulation must be taken into account, especially for the lower bracket design. In the context of domestic pure electric vehicles, lightweight design typically involves lowering the thickness of the bracket bottom and incorporating lightweight apertures underneath the bracket to achieve desired outcomes, as illustrated in Fig. 1. Our topology majorization design is on the basis of these principles. For the design of a pure electric vehicle battery pack system in China, Rhino 6.0 software developed by Robert McNeel Inc. was utilized. Aside from that, Altair Inspire 2022 software developed by Nasdaq: ALTR was employed for finite element analysis and topology betterment design of the same battery pack system.

### Manufacturing and analytical methods

As this design is aimed at developing high-performance lightweight battery bracket products, it falls within the realm of small-batch part production during the product development stage. Traditional manufacturing methods such as machining, casting, and welding would immensely escalate costs. As a result, this paper predominantly relies on 3D printing to manufacture such intricate parts. To this end, the Lite600 industrial high-precision 3D printer manufactured by Shanghai Union Tech 3D Technology Co., Ltd. was utilized. Above all, the original model and manually reconstructed model were saved in STL format. Afterwards, they were imported into Materialise Magics 21.0, a 3D printing data processing software, so as to carry out printing parameter configuration. During the above process, relevant printing parameters were specifically set as follows: the molding material was white photosensitive resin, with a filling density of 100%. The printing temperature was maintained at a constant 24 °C, and the printing layer thickness was set to 0.08 mm.

The 3D printed brackets, housings, and lightweight battery brackets underwent surface treatment consisting of several steps. First and foremost, support removal was carried out, followed by rough polishing using sandpaper. Finally, the components were polished with a polishing cloth. Upon completion of the surface treatment process, the finished battery pack system components were assembled to verify the fit.

## Results and discussion

### Strength analysis of the lower battery tray bracket for a electric vehicle

#### Methods of analysis

**Figure 2:**
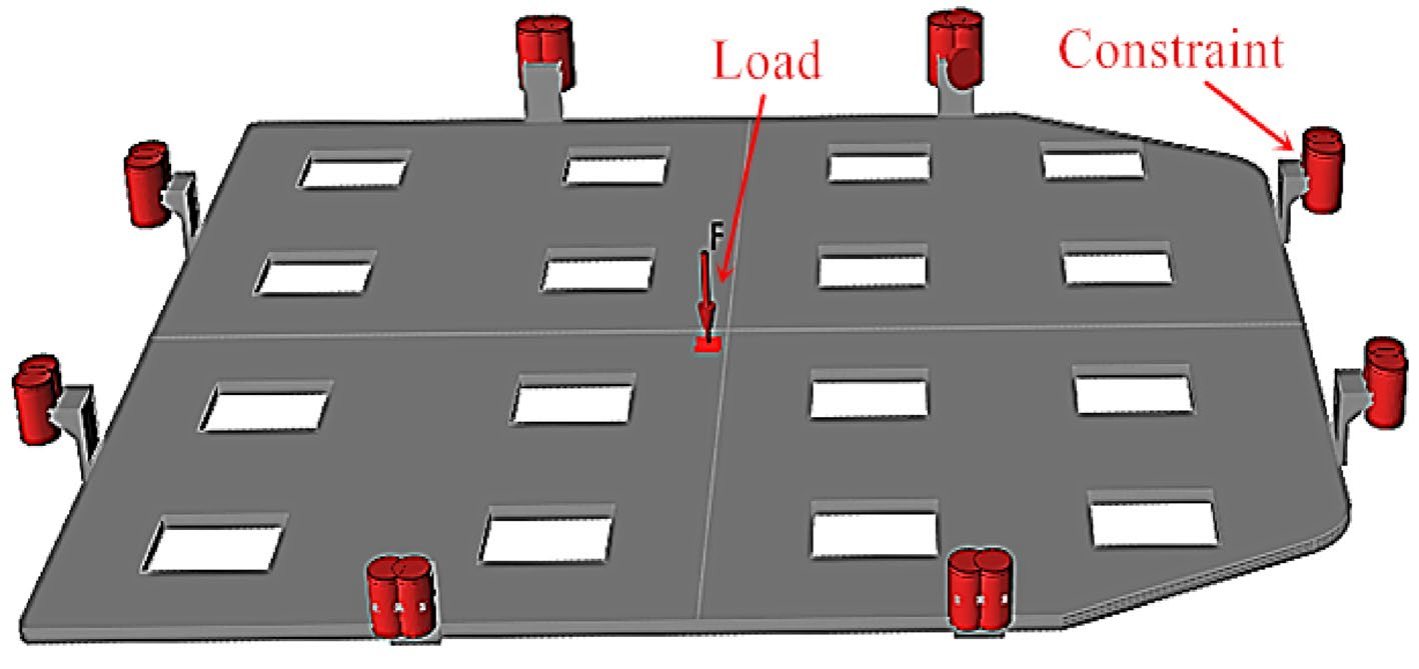
Load and restraining position of a battery tray of an electric vehicle.

For the convenience of analysis, the designed lower bracket model was scaled down by a factor of 0.2. The strength analysis of the lower bracket is conducted by adopting Inspire software. Specific simulation parameters are as follows: the units are set to millimeters, kilograms, newtons, and seconds after importing the parts, and the analysis material is aluminum alloy Al 6061. Since the force on the battery bracket mainly originates from the battery, and the weight of the battery in this model is approximately 100 kg, it is pivotal to ensure the reliability of the battery installation. As a consequence, the force and deformation of the battery bracket under typical working conditions, such as bumpy roads and sharp turns, need to be investigated systematically and comprehensively. To simulate the load-bearing capacity of the battery bracket under bumpy road conditions, a surface load of 5 times the gravity of the battery is applied perpendicular to the bottom surface of the bracket (Z-axis direction). Given that the model is scaled down by a factor of 0.2, the load is approximately 980 newtons. The fixed holes are constrained, and "more accurate" calculation speed/precision is selected for single load analysis. The size of the analysis unit is set to 5 mm, as displayed in Fig. 2.

#### Analysis results

**Figure 3:**
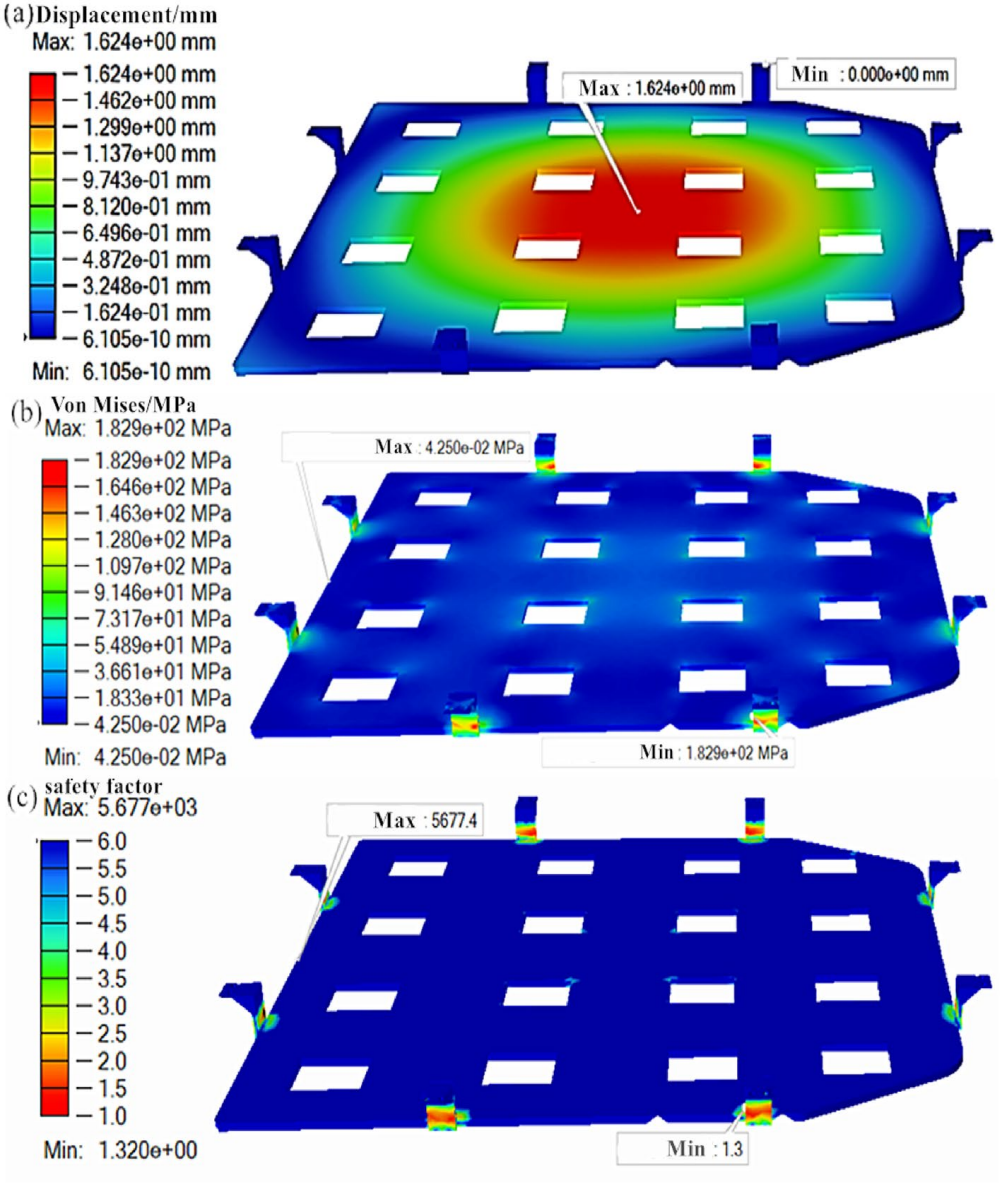
Force analysis results of battery carrier: (**a**) displacement cloud map; (**b**) stress cloud map; (**c**) Safety factor.

On the basis of the aforementioned analysis parameter settings, the initial model of the lower battery tray bracket is imported into Altair Inspire software for initial strength analysis, and the results are depicted in Fig. 3. Upon observation, it is noted that the maximum displacement of the lower tray bracket is 1.62 mm, with the highest displacement occurring at the center of the battery bracket, in accordance with the displacement distribution pattern. The maximum Mises equivalent stress is 182.90 MPa, illustrating some inhomogeneous stress distribution, with the highest stress concentrated mainly in the upward folded lug part of the battery bracket. Furthermore, the minimum safety factor exceeds 1.3, the measured mass value is 0.685 kg, and the elastic modulus is 1.11 MPa, all of which meet the design strength requirements. Considering the safety factor and modulus of elasticity, there is still significant potential for mass reduction.

### Topology optimization design of the lower battery tray bracket

#### Methods of analysis

**Figure 4:**
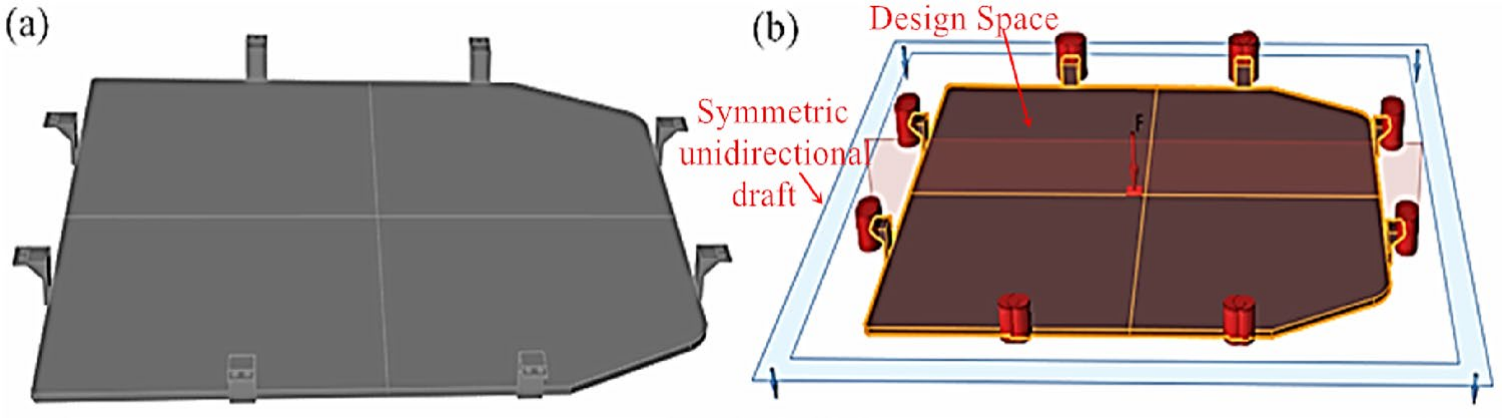
Topology optimization parameter settings: (**a**) Filled model; (**b**) Load and constraint settings.

In an effort to broaden the design possibilities of the lower bracket of the battery tray for new energy vehicles, it is highly essential to pre-fill the lightweight holes in the lower bracket of the battery tray before conducting topology optimization design. The same parameters for force analysis are set for the lower tray bracket before and after topology betterment. Within Altair Inspire software, the part of the battery tray excluding the fixed holes is designated as the design space, highlighted in red in Fig. 4, while the remaining parts are considered non-design space, indicated in gray in Fig. 4. To materialize optimal topology majorization results, shape control is applied to the battery tray section. Considering the shape characteristics of this model, symmetric + unidirectional pull-out constraints are set. The optimization objective is defined as maximizing stiffness, with a mass objective of 30%, and a thickness constraint of 5 mm for optimization, as illustrated in Fig. 4.

#### Analysis results of topology optimization

**Figure 5:**
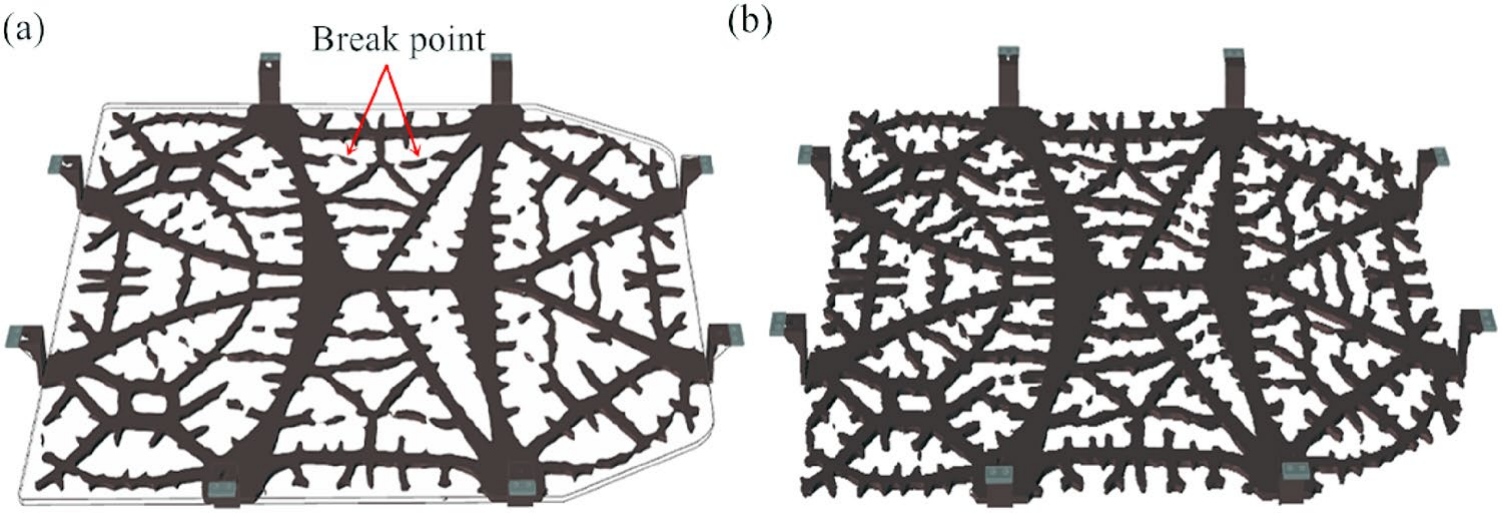
Topology optimization results of battery tray: (**a**) before adjustment; (**b**) after adjustment.

The results of the topology betterment for the tray bracket under the aluminum alloy battery are depicted in Fig. 5, from which we observe that the tray exhibits a dendritic structure after topology betterment, with many areas remaining disconnected. Despite attempts to optimize these defects by adjusting the smoothing result slider, it is found to be ineffective, resulting in difficulties in performing PolyNURBS fitting. Moreover, manual reconstruction is not feasible on account of the complexity inherently existing in the model. For this reason, how to effectively address the challenges correlated with the reconstruction of the model post-topology optimization while ensuring its processability remains a current barrier in the lightweight design of battery pack trays for new energy electric vehicles.

### Reconstruction solution for topology optimized parts

**Figure 6:**
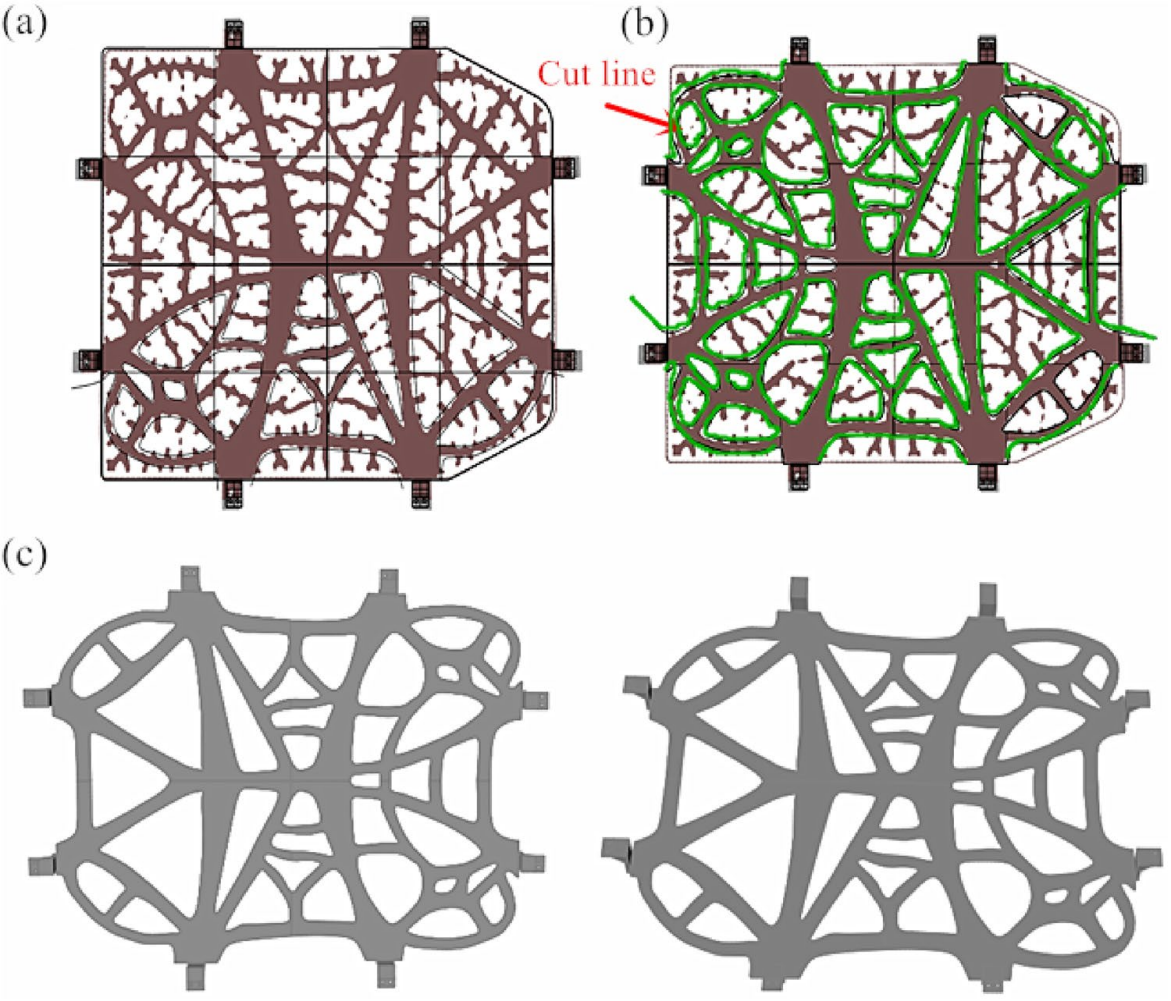
Reconstruction of topology optimization model: (**a**) importation of images and models; (**b**) division of cutting lines; (**c**) reconstruction effect.

In line with a further analysis on the structure of the pallet bracket under the battery after topology optimization, the pallet bracket exhibited a dendritic shape, with intertwining branches. Conventional inverse reconstruction methods proved ineffective in achieving the desirable reconstruction. Following the research, an image-based reverse reconstruction method was put forth accordingly. This method involves exporting the topology optimized model as an image and employing a cutting technique in other 3D software to remove the branches while retaining the trunks. This approach ensures that the topology optimized model remains processable and allows for redesign if necessary. To verify the effectiveness and feasibility of this method, the exported image and model are imported into the 3D modeling software Rhino 6 simultaneously for scribing and cutting. Boolean operations are subsequently employed to perform the cutting, bringing about the effect depicted in Fig. 6, from which we can learn that the reconstructed battery bracket features a clear structure. The lower part of the bracket can be manufactured through stamping, while the lugs can be produced via milling or stamping. Welding can be used to connect the bracket and lugs, facilitating mass production in line with the enterprise's requirements.

### Strength calibration of topology optimization model

#### Method of analysis

**Figure 7:**
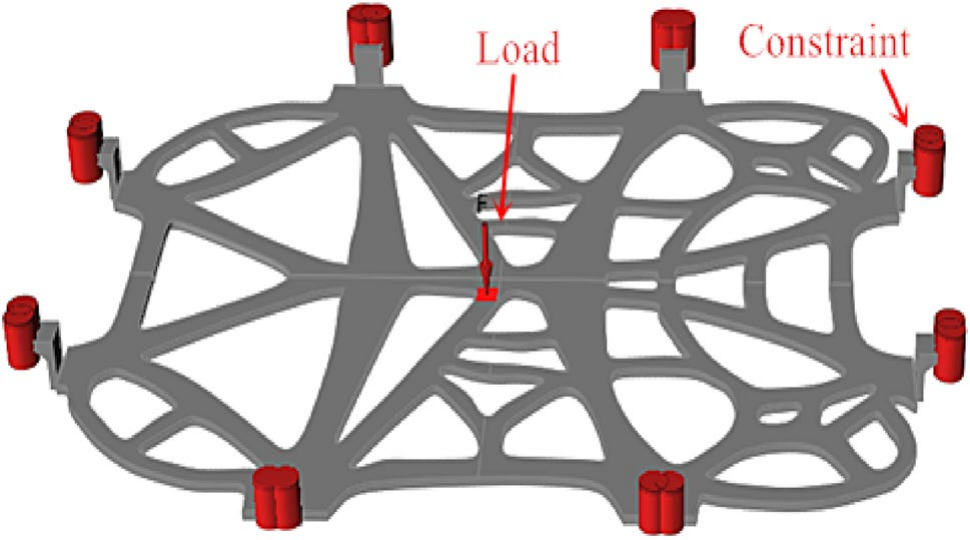
Load and constraint position of a battery topology optimization tray for an electric vehicle.

To facilitate the analysis, the reconstructed model of the battery tray bracket was scaled down by 0.2 times. Topology optimization for the strength analysis of the battery tray bracket was carried out by employing Inspire software. Specific simulation parameters included setting the unit to mm kg N s after importing the parts, with aluminum alloy Al 6061 chosen as the analysis material. The force exerted on the battery tray bracket principally originates from the battery, with an estimated weight of 100 kg in the model. To ensure the reliability of the battery installation, it is imperative to probe deep into the force and deformation of the battery tray bracket under typical working conditions such as bumpy roads and sharp turns. For simulating the load-bearing conditions of the battery tray bracket under bumpy road conditions, a surface load equivalent to 5 times the gravitational force of the battery was applied perpendicular to the bottom surface of the tray (Z-axis direction). Given the model's scaling factor of 0.2, the load amounted to approximately 980 N. The load was constrained at fixed holes, with calculation speed/precision set to "more accurate", and the working condition selected as single load condition analysis. The analysis unit size was set to 4 mm, as exhibited in Fig. 7.

#### Analysis results

**Figure 8:**
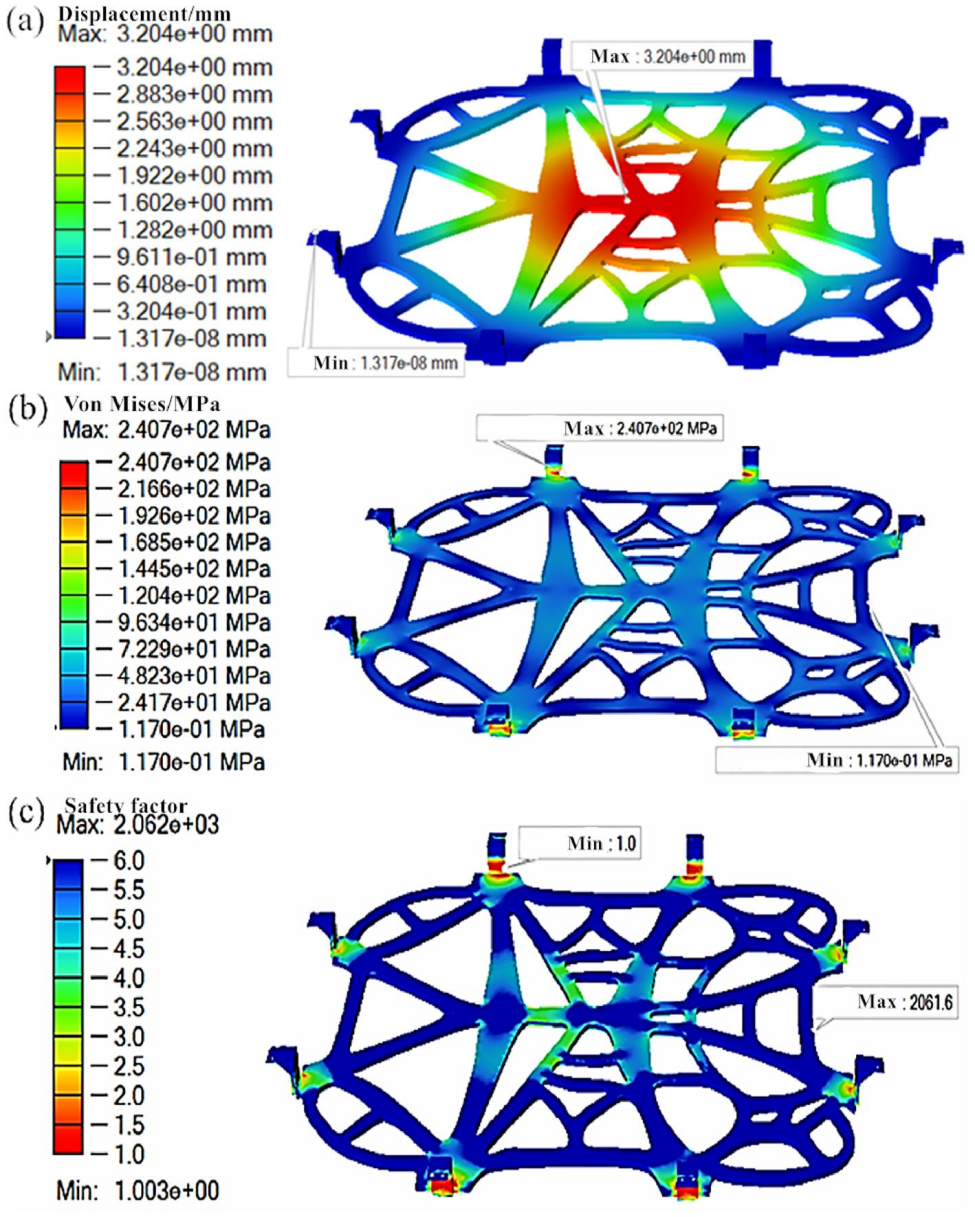
Force analysis results of the battery carrier: (**a**) Displacement cloud map; (**b**) stress cloud map; (**c**) safety factor.

On the basis of the aforementioned analysis parameter settings, the initial model of the battery pack lower tray bracket was imported into Altair Inspire software for strength assessment, with its results illustrated in Fig. 8, from which we can learn that the tray bracket below the battery pack can achieve a maximum displacement of 3.20 mm. This represents an increase in comparison with the pre-topology betterment. Nevertheless, it remains within an acceptable range. Furthermore, the maximum Mises equivalent force is measured at 240.7 MPa, which marks an increase from pre-topology optimization. Nonetheless, it exhibits a more uniform distribution at the bottom. The minimum safety factor is set as 1, meeting the design requirements. After topology optimization, the mass of the bracket is recorded as 0.348 kg, which apparently suggests a reduction of 50.8% compared with its pre-optimization state. Of particular note is that the initial mass of the bracket before scaling was 85.63 kg, exhibiting a 50.8% decrease post-optimization. This translates to a reduction of 42.07 kg in the bracket mass. The modulus of elasticity is measured at 0.75 MPa after topology optimization, suggesting a 67.6% decrease compared to its pre-optimization state. This augment in impact resistance of the battery bracket is notable. The topology optimized design of the battery tray under the battery pack aims to minimize overall mass while ensuring strength and safety performance. Apart from that, it ensures that manufacturing costs remain within a reasonable range, thereby achieving a balance between safety and economic considerations.

### Assembly analysis of topology optimized model

**Figure 9:**
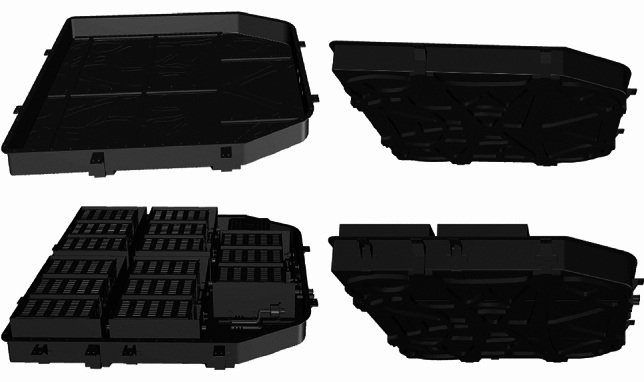
Assembly effect of battery pack, tray and bracket.

In the assembly process, the geometrically reconstructed model was first exported to “.stp format” by adopting Altair Inspire software. Afterwards, the reconstructed model was imported into Rhino 6 software, where the optimized battery lower tray bracket replaced the lower tray bracket of the original model for assembly. Just as exhibited by the assembly effect depicted in Fig. 9, the designed battery lower tray and bracket are closely matched with each other. After conducting a thorough check for assembly conflict, it was confirmed that no conflicts exist between the structures. The fixed bracket can be seamlessly connected to the battery lower tray bracket through welding. What's more, the battery tray bracket can be mass-produced through stamping. This manufacturing approach not only meets the enterprise's requirements for weldability, corrosion resistance, and impact resistance, but also satisfies the needs for automation and mass production.

### 3D Printing and assembly verification of topology optimized models

#### Data processing of 3d printed parts

**Figure 10:**
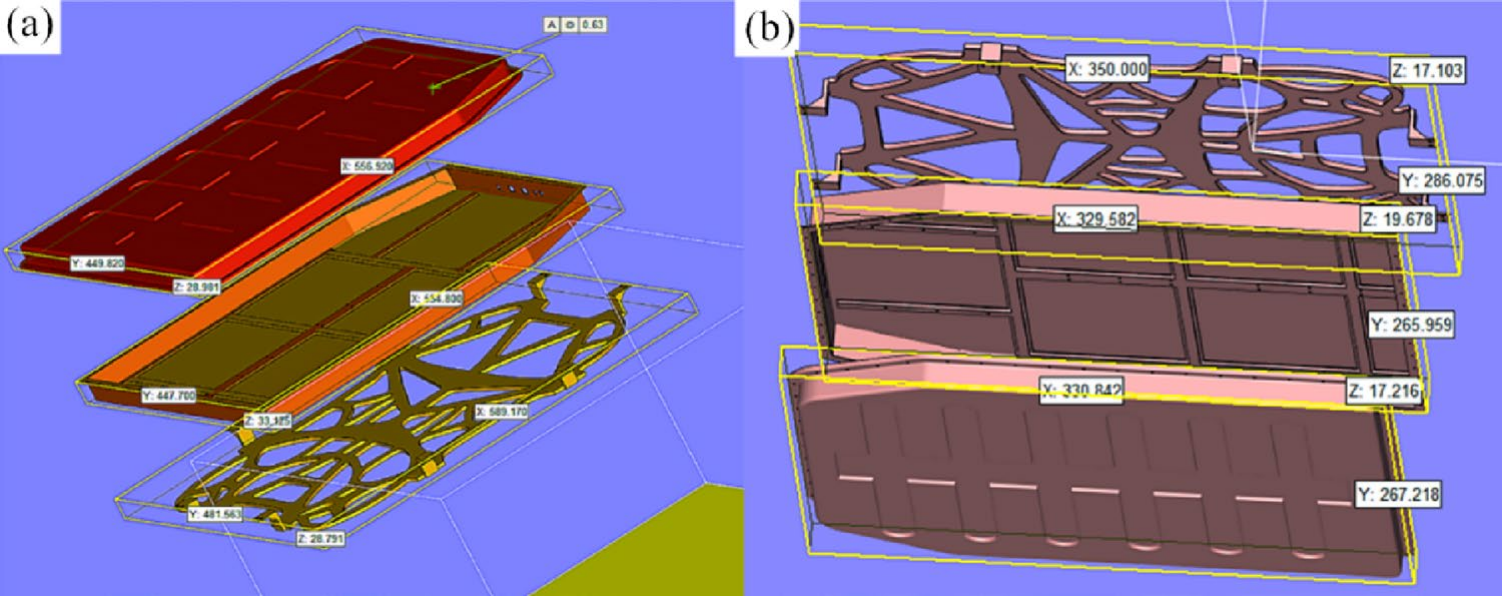
Data processing of 3D printed parts: (**a**) Original effect; (**b**) effect after adjusting the placement.

In some sense, the use of 3D printing technology in part processing can tremendously lessen the product development cycle and lower associated costs. During the process of 3D printing, different methods employed for part placement and support addition can trigger varying amounts of support and molding layer thickness, which may exert direct influence on the quality and efficiency of part production. More importantly, the battery pack system parts were initially imported into Materialise Magics 22 software, as depicted in Fig. 10a. Based upon previous research on 3D printing part placement and support addition methods^15^, a tilted vertical placement approach was adopted for the battery pack upper tray, lower bracket, and lower tray bracket. To be specific, an angle of 75° between the molded part and the substrate was implemented to minimize the need for excessive support addition, particularly in critical areas such as the inner sections of the upper tray, lower bracket, and lower tray bracket, as illustrated in Fig. 10b. This strategy aims to avoid unnecessary support addition, as exhibited in Fig. 10b.

#### Printed part effect and assembly inspection

**Figure 11:**
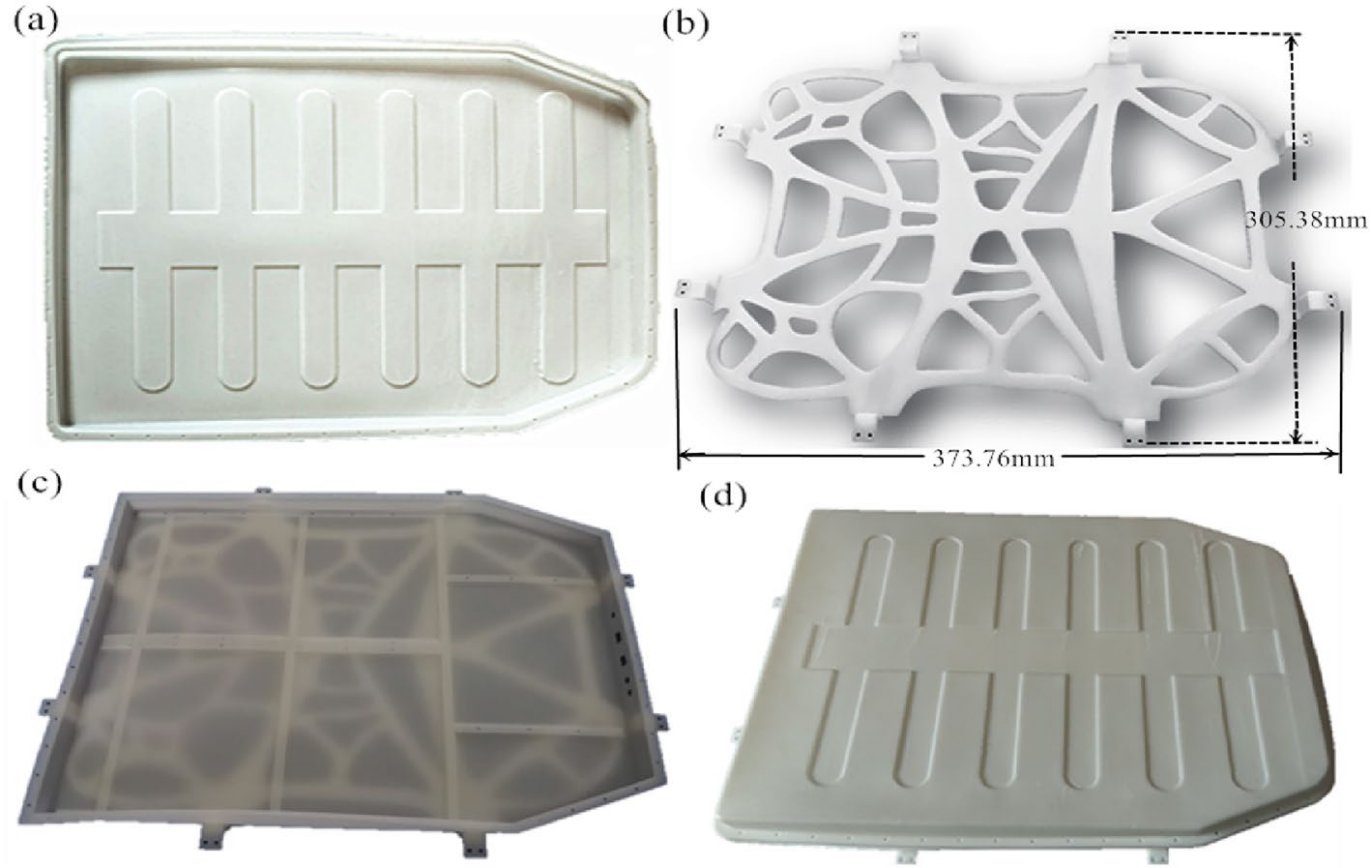
Analysis of 3D printing battery packaging: (**a**) Upper tray; (**b**) Lower tray; (**c**) Lower tray bracket; (**d**) Overall assembly effect.

The final model effect of 3D printing is shown in Fig. 11. Observing Fig. 11a,b, it is found that the surface of the battery pack tray and bracket components is bright and has low roughness after 3D printing is completed. To be more specific, no conspicuous overhanging slag is found on the surface. Moreover, there are no discernible warping or deformation defects. While some support was added in certain areas such as the fixed holes, which may slightly affect the surface finish. But it remains within acceptable limits. Subsequently, the finished part is detached from the substrate, while post-processing tasks such as support removal, polishing, sanding, elimination of surface burrs, and cleaning with alcohol are performed to actualize the final part model.

As clearly demonstrated by the assembled 3D-printed battery pack tray and bracket exhibited in Fig. 11c,d, the above two parts fit closely with each other, with no apparent assembly conflicts between these two parts. This observation indicates that the dimensional accuracy and alignment of the designed parts meet the specified requirements.

## Conclusion

1. The maximum displacement of the tray bracket under the topology optimized battery is 3.20 mm, which is higher than pre-topology optimization. Nevertheless, the degree of betterment is not so satisfactory as we might expect. The maximum Mises equivalent force is 240.7 MPa, which is higher than pre-topology optimization, but this force is more evenly distributed at the bottom. The minimum safety factor of 1 equals to 1, meeting the design requirements. The mass of 0.348 kg is 49.2% lower than pre-topology optimization. The maximum displacement of the battery bracket under the topology optimized battery is also 3.20 mm, which is lower than pre-topology optimization, by 49.2%.
2. The geometrically reconstructed battery bracket exhibits a clear structure. The lower part of the bracket can be manufactured by stamping, while the lugs can be produced through milling or stamping processes. Welding can be utilized for connecting the bracket with the lugs, thus fulfilling the requirements for mass production within the enterprise.
3. The battery pack tray and bracket parts manufactured through 3D printing can give birth to bright surfaces with ultra-low roughness. To put it another way, no evident dregs, warping, deformation, or other defects can be observed on the printed surfaces. Upon assembly, the battery pack tray and bracket printed by 3D technology demonstrate close alignment with each other, without any apparent assembly conflicts between the components.

Certainly, to strengthen the all-round performance of the battery pack system for new energy electric vehicles, further experiments are essential. These may include 3D printing of high-performance cooling water circuits for batteries, assessing the impact resistance of battery systems, and other relevant studies. These endeavors aim to establish the groundwork for the optimized design and mass production high-performance lightweight battery pack systems.

## Data Availability

The datasets used and/or analyzed during the current study are available from the corresponding author on reasonable request.
